# Development of a fast and efficient root transgenic system for functional genomics and genetic engineering in peach

**DOI:** 10.1038/s41598-020-59626-8

**Published:** 2020-02-18

**Authors:** Shengli Xu, Enhui Lai, Lei Zhao, Yaming Cai, Collins Ogutu, Sylvia Cherono, Yuepeng Han, Beibei Zheng

**Affiliations:** 1 CAS Key Laboratory of Plant Germplasm Enhancement and Specialty Agriculture, Wuhan Botanical Garden, The Innovative Academy of Seed Design, Chinese Academy of Sciences, Wuhan, 430074 China; 2 University of Chinese Academy of Sciences, 19A Yuquanlu, Beijing, 100049 China; 3 Sino-African Joint Research Center, Chinese Academy of Sciences, Wuhan, 430074 China

**Keywords:** Gene delivery, Plant biotechnology

## Abstract

Peach is an economically import fruit crop worldwide, and serves as a model species of the Rosaceae family as well. However, peach functional genomics studies are severely hampered due to its recalcitrance to regeneration and stable transformation. Here, we report a fast and efficient *Agrobacterium rhizogenes*-mediated transformation system in peach. Various explants, including leaf, hypocotyl and shoot, were all able to induce transgenic hairy roots, with a transformation efficiency of over 50% for hypocotyl. Composite plants were generated by infecting shoots with *A. rhizogenes* to induce transgenic adventitious hairy roots. The composite plant system was successfully used to validate function of an anthocyanin-related regulatory gene *PpMYB10.1* in transgenic hairy roots, and two downstream genes, *PpUFGT* and *PpGST*, were strongly activated. Our stable and reproductive *A. rhizogenes*-mediated transformation system provides an avenue for gene function assay, genetic engineering, and investigation of root-rhizosphere microorganism interaction in peach.

## Introduction

Peach (*Prunus persica*) is one of the most widely cultivated fruit trees in temperate and subtropical zones throughout the world. In addition to its agricultural and economic importance, peach also serves as a model species of the Rosaceae family due to its small genome size of approximately 230 Mb/haploid^1,2^. The high-quality draft genome sequence of doubled haploid peach cv. Lovell^1^ has facilitated the identification of genes responsible for agronomic importance traits using forward and/or reverse genetics strategies. However, gene function analysis is severely hindered by lack of a transformation protocol in peach. The key obstacle in generating transgenic peach plants is attributed to strong recalcitrance of adult tissues to somatic embryogenesis^3–6^.

Development of a reliable regeneration system for somatic tissue is crucial for the improvement of woody fruit species by transgenic approaches^7^. Callus induction is the first step in the development of a transformation protocol as callus is usually used for genetic transformation and adventitious shoot regeneration^8–10^. A variety of explants, such as leaf, stem and calyx, have been successfully used to induce callus production in peach^11^. However, browning due to oxidation of polyphenols causes loss of regenerative ability and subsequent cell death, thus, it represents a major limitation of callus culture in woody plants^12,13^.

*Agrobacterium tumefaciens*-mediated transformation has been developed in several fruit species of *Prunus*, including Plum (*P. domestica*)^14^, Japanese Apricot (*P. mume*)^15^, Chinese Plum (*P. salicina*)^16^ and Apricot (*P. armeniaca*)^17^. *A. tumefaciens*-mediated transformation has also been used to transfer visible marker genes encoding green fluorescent protein (GFP) or *β*–glucuronidase (GUS) to peach^5,18,19^. However, the transformation efficiency was low, and a reliable and reproducible transformation system is yet to be developed. Many plant species are resistant to development of an efficient transformation methodology due to the recalcitrance of somatic tissues to regeneration and/or low efficiency of *A. tumefaciens*-mediated transformation. Gene function assay can be carried out with transgenic tissues instead of transgenic plantlets. Moreover, methods for transgenic tissue production have advantages in comparison to whole plant transformations as transgenic tissues can be produced rapidly and inexpensively. *A. rhizogenes*-mediated tissue production, an alternative method for *A. tumefaciens*-mediated transformation, is often used to induce adventitious root development, i.e. hairy roots, at the site of wounding and infection^20,21^. The ability of *A. rhizogenes* to induce the formation of neoplastic and plagiotropic roots is attributed to its extrachromosomal replicon root-inducing (Ri) plasmid. T-DNA of binary vector carrying the gene(s) of interest co-transfers with Ri plasmid to integrate exogenous genes into plant genome^22,23^. As each hairy root generated from a single cell represents an independent transformation event, *A. rhizogenes*-mediated transformation is a high-throughput method to induce transgenic hairy root lines within a short time.

A variety of explants such as leaf and stem have been used to induce hairy roots by *A. rhizogenes* infection to validate function of genes involved in root-rhizosphere interaction^24,25^, secondary metabolism^26^, and resistance to biotic and abiotic stresses^12,27^. Transgene overexpression or RNA interference (RNAi) in *A. rhizogenes*-transformed roots have been proven to be an invaluable approach in soybean^28^, brassica^29^, tomato^30^, beet^31^, citrus^32^ and apple^33^. As mentioned above, regenerated roots are induced from single cells, which ensures that they are non-chimeric^34^. Thus, trangenic hairy roots are an ideal explants to generate non-chimeric transgenic calli with vigorous growth, which can serve as an alternaive system for gene funciton assay in peach.

In this study, an efficient method for hairy root induction in peach has been established based on *A. rhizogenes*-mediated transformation. To easily demonstrate the success of transformation protocol, a red fluorescent protein (DsRED1) was utilized as a visual marker to distinguish between transgenic and non-transgenic roots. In addition, we further demonstrated that over-expression of the peach anthocyanin-related R2R3-MYB transcription factor *PpMYB10.1*^35^ results in massive accumulation of anthocyanidins in transgenic roots. Our root transgenic method has advanced and opened an avenue for gene function assay via over-expression and/or knock-out of various genes of interest.

## Results

**Figure 1:**
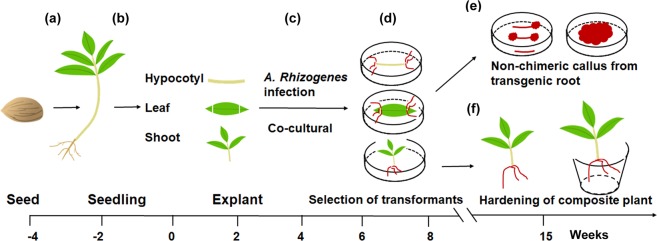
Procedure for *Agrobacterium rhizogenes*-mediated transformation of peach. (**a**) Seeds were sterilized and soaked in sterilize distill water overnight, and then germinated in MS medium. (**b**) Hypocotyl and leaf explants were cut into 10-mm segments and then immersed in the suspension of *A. rhizogenes* strain MSU440 containing *DsRED1* as selectable marker, whereas, shoot explants were infected by immersing cut site in the *A. rhizogenes* suspension. (**c**) Co-cultural of infected plants and agrobacteria for 3 days on MS medium with acetosyringone under darkness. (**d**) Transfer the co-cultural explants into a new MS medium containing 400 mg/L cephalosporin, and then validate the transgenic hairy roots by screening red fluorescent signal of DsRED1. (**e**) Transgenic hairy roots from hypocotyl and leaf explants were used to induce non-chimeric callus. (**f**) Composite plants with transgenic hair roots were transferred to plot after 2-week hardening.

The pipeline steps of *A. rhizogenes*-mediated hairy root transformation in peach are shown in Fig. 1. Briefly, mature seeds of ‘Shengli’ were surface-sterilized to germinate on MS medium (Fig. 1a). Hypocotyls and fully expanded leaves of 4-week-old seedlings were used as transformation explants (Fig. 1b,c). Approximately one month after infection, the transgenic roots were proved to be positive by screening for DsRED1 fluorescence signal (Fig. 1d). Transgenic hairy roots from leaves and hypocotyls could be used to induce positive transgenic calli (Fig. 1e). The composite plants with fluorescent roots were hardened and subsequently transferred to pots (Fig. 1f).

**Figure 2:**
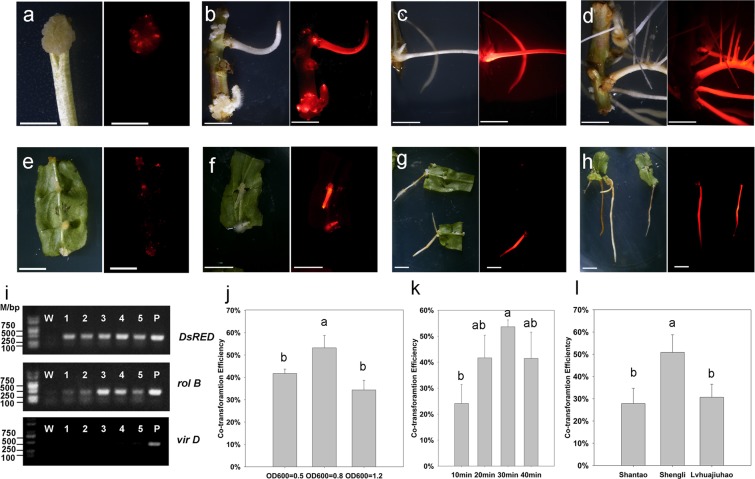
*A. rhizogenes*-mediated induction of transgenic hairy roots and selection of optimal infection conditions. (**a**) Chimeric callus tissues induced from hypocotyl explants. (**b**–**d**) Transgenic hairy roots from hypocotyl explants three, four, and six weeks after infection, respectively. (**e**) Chimeric callus tissues induced from leaf explants. (**f**–**h**) Transgenic hairy roots from leaf explants three, four, and six weeks after infection, respectively. (**i**) Validation of transgenic hairy roots using PCR analysis, the full-length gels were shown in Figure S2(a–e). (**j**) Co-transformation rate of various infection concentrations (OD_600nm_) with 30 min infection duration. (**k**) Co-transformation rate of various infection durations (min) with infection OD_600nm_ = 0.8. (**l**) Co-transformation rate of various varieties (30 min infection duration and OD_600nm_ = 0.8). The left and right images in a-h were captured using bright field and fluorescence microscopy, respectively, with a scale bar of 1 cm. Hypocotyl was the infection explant in (**j**–**l**), different lowercase letters in (**j**–**l**) indicate statistically significant difference (*P* < 0.05).

Hypocotyl was cut at a slant angle into small segments to increase the surface area for bacterial infection (Fig. 2a). Two weeks after infection, callus tissues appeared around the slanting cut site and expanded gradually. One more week later, adventitious hairy roots regenerated from callus tissues (Fig. 2b,c). The hairy roots grew vigorously within the following one month (Fig. 2d). With the same regenerate progress, leaves were also collected as explants to generate hairy roots (Fig. 2e–h). The hairy roots regenerated from hypocotyls were strong and showed vigorous growth (Fig. 2d), however, those from leaves were slightly slim (Fig. 2h). Overall, hypocotyl was a better explant than leaf for hairy root regeneration in peach.

DsRED1 fluorescence was detected in transgenic hairy roots. PCR analysis showed that transgenic hairy roots with DsRED1 fluorescence had the expected DNA fragment of *DsRED1* with 443 bp in size, but not for non-transformed roots (Fig. 2i). Likewise, the expected DNA fragment with the size of 450 bp from the *rol* B gene responsible for directing differentiation of roots was also detected for transgenic hairy roots with DsRED1 fluorescence, but absent for non-transformed roots. In addition, the expected DNA fragment with the size of 441 bp from the *vir* D gene located outside of T-DNA region of Ri plasmid was only detected for the *A. rhizogenes* strain, but absent for both transgenic and non-transgenic roots. These results indicated that transgenic hairy roots contained the target gene and had no contamination of *A. rhizogenes*. Taken together, these results demonstrated a successful development of a stable root transformation system in peach.

Hypocotyls of ‘Shengli’ were used as explants to screen optimum infection conditions. Firstly, different infection durations (10, 20, 30, and 40 min) were tested with the optimized *A. rhizogenes* concentration of OD_600nm_ = 0.8 as reported in plum^27^. When infection durations ranged from 10 to 30 min, co-transformation efficiency increased accordingly, with mean values of 24.12 ± 7.30%, 41.67 ± 8.71%, and 53.63 ± 2.63% in 10, 20, and 30 min, respectively (Fig. 2k). However, co-transformation efficiency decreased to 41.47 ± 10.11% at the 40-min infection duration. Statistical analysis revealed that co-transformation efficiency in 30 min was significantly higher than that in 10 min. Thus, the optimum infection time was deemed to be 30 min.

Subsequently, various *A. rhizogenes* concentrations (OD_600nm_ = 0.5, 0.8 or 1.2) were also evaluated with the optimized infection time of 30 min. Co-transformation efficiency increased significantly from OD_600nm_ = 0.5 (41.73 ± 1.95%) to OD_600nm_ = 0.8 (53.22 ± 5.55%), but decreased significantly when OD_600nm_ = 1.2 (38.70 ± 9.22%) (Fig. 2j). This suggested that *A. rhizogenes* concentration of OD_600nm_ = 0.8 was the optimum infection concentration in peach, consistent with that reported in plum^27^. Taken together, these results indicated an optimum *A. rhizogenes* infection condition of OD_600nm_ = 0.8 and 30 min duration.

To evaluate potential effect of genotypes on hairy root transformation efficiency, hypocotyls of two additional varieties, ‘Shantao’ and ‘Lvhuajiuhao’, were testified using the optimum infection condition mentioned above. As shown in Fig. 2l, co-transformation efficiency for ‘Shengli’ (50.86 ± 7.93%) was significantly higher than those for ‘Shantao’ (27.83 ± 6.88%) and ‘Lvhuajiuhao’ (30.68 ± 5.87%). This suggested an impact of peach genotypes on co-transformation efficiency.

Positive transgenic roots containing the *DsRED1* gene can be easily confirmed by screening fluorescence signal of the DsRED1 protein at early stages of transformation. Regenerated calli appeared around the cut site of hypocotyl segment two weeks after infection, but not whole tissues had red fluorescence (Fig. 2a). This suggested a chimera formed by untransformed cells. One more week later, adventitious hairy roots appeared and had strong red fluorescence (Fig. 2b). This red fluorescence was still easily detectable after one week (Fig. 2c) and even after 1.5 month of growth (Fig. 2d).

**Figure 3:**
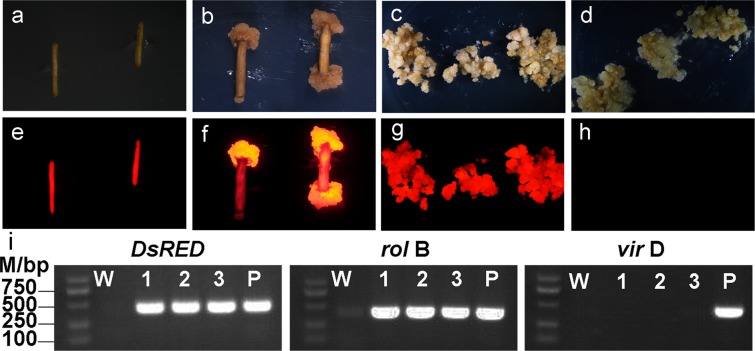
Detection of red fluorescence signal of DsRED1 in co-transformed hair roots and callus tissues using bright filed (above) and fluorescence microscopy (bottom). (**a**,**e**) Transgenic hairy roots carrying the *DsRED1* gene. (**b**,**f**) Non-chimeric callus tissues induced from transgenic hairy roots carrying the *DsRED1* gene. (**c**,**g**) Propagation of non-chimeric transgenic callus tissues. (**d**,**h**) Wild type callus tissues without red fluorescence signal are used as negative control. (**i**) PCR validation (Lower) of non-chimeric callus tissues from transgenic hairy roots carrying *DsRED1*, the full-length gels were shown in Fig. S2(d).

To rapidly propagate transgenic tissues, transgenic hairy roots were utilized to induce callus. One or two months after infection, hairy roots with red fluorescence were collected for callus induction (Fig. 3a,e). After two weeks of induction, non-chimeric callus tissues with strong fluorescent signal were observed (Fig. 3b,f), and showed a vigorous growth with ability to propagate rapidly (Fig. 3c,g). After more than one year of subculture, the callus tissues still had strong non-chimeric fluorescent signal and contained the DsRED1 DNA fragment as proven by PCR analysis (Fig. 3i). Wild type callus was used as negative control, which did not show any fluorescent signal (Fig. 3d,h). Thus, a non-chimeric callus induction system was successfully established using transgenic hairy root as explant in peach.

**Figure 4:**
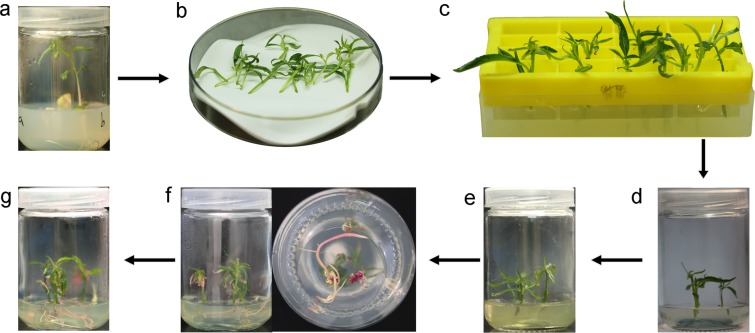
Flow chart for production of composite plants overexpressing *PpMYB10.1* in transgenic hair roots. (**a**) Seedlings for infection. (**b**) Preparation of wild type shoots by removing roots. (**c**) Infection of shoots with *A. rhizogenes*. (**d**) Co-cultural of *A. rhizogenes* and shoots in MS medium. (**e**) Selection of transformants in MS medium containing kanamycin (50 mg/L) and cephalosporin (400 mg/L). (**f**) Regeneration of transgenic callus tissues and hairy roots from the cut site. (**g**) Composite plants overexpressing *PpMYB10.1* in transgenic hair roots.

**Figure 5:**
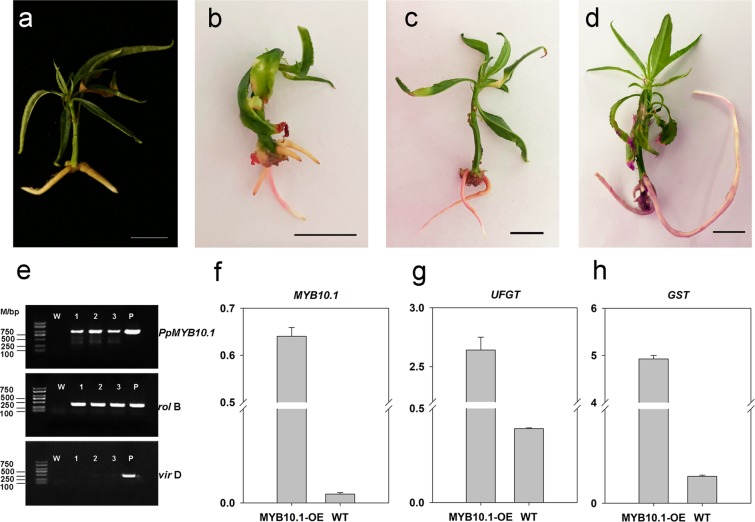
Phenotype and molecular validation of transgenic composite plants over-expressing *PpMYB10.1* in roots. (**a**) Adventitious roots induced by auxin from wild-type shoots. (**b**–**d**) Anthocyanin pigmentation in hairy roots of transgenic composite plants. Scale bar: 1 cm. (**e**) Validation of transgenic hairy roots overexpressing *PpMYB10.1* by PCR analysis, the full-length gels were shown in Fig. S2(e–g). Expression levels of *PpMYB10.1* (**f**), *PpUFGT* (**g**) and *PpGST* (**h**) in transgenic and wild-type roots.

Since both hypocotyl and leaf explants were successfully used to induce *A. rhizogenes*-mediated adventitious hairy root, transformation protocol was advanced to produce composite plant with wild-type shoot and transgenic root (Fig. 1). This composite plant system was used to testify the function of *PpMYB10.1*, a key regulator of anthocyanin coloration in peach flesh^31^ via its overexpression in hairy roots. Binary vector pSAK277 carrying the *PpMYB10.1* gene was introduced to shoot tissues using *A. rhizogenes*-mediated transformation system developed in this study (Fig. 4). Two weeks after infection, red callus tissues were clearly visible around cut sites of shoots (Fig. 4f). Two more weeks later, adventitious hairy roots with pink color were regenerated from red callus (Fig. 4f). After two or three months of growth, transgenic hairy roots were large enough to support the shoots (Fig. 4g). Anthocyanin pigmentation was clearly observed in roots of composite plants overexpressing *PpMYB10.1* (Fig. 5b–d), while white coloration was observed in adventitious roots induced by auxin from wild-type shoots (Fig. 5a). PCR and qRT-PCR were performed to confirm overexpression of *PpMYB10.1* in hairy roots. As a result, the expected fragment of *PpMYB10.1* was detected for pink-colored hairy roots, but absent for wild-type roots (Fig. 5e). Expression level of *PpMYB10.1* was over 100-fold higher in pink-colored hairy roots than that in wild-type roots (Fig. 5f). Likewise, two structural genes, *PpUFGT* and *PpGST*, were also activated, and their expression levels were approximately 10- and 100-fold higher, respectively, than those in wild-type roots (Fig. 5g,h). However, other structural genes, such as *PpCHS*, *PpDFR* and *PpF3’H*, showed no difference in expression levels between transgenic and wild-type roots (Fig. S1). These results indicated that anthocyanin-related regulatory gene *PpMYB10.1* was able to activate anthocyanin biosynthesis gene *PpUFGT* and transport gene *PpGST*, resulting in anthocyanin accumulation in transgenic hairy roots of peach.

## Discussion

A variety of economically important fruit trees, such as apple, peach, pear, cherry, quince, apricot, raspberry, and strawberry, plum, almond and loquat, belong to the Rosaceae family. Analysis of taxonomic diversification within this family has proposed that three species, apple, peach and diploid strawberry, can serve as model systems for genomics studies in Rosaceae. Genomics study has been extensively conduced in peach and a number of genes responsible for important traits, such as fruit coloration^36–38^, texture^39–41^, sugar and acid contents^42^, and fruit size^42^, have been identified. However, their function analysis was usually validated in model plants such as *Arabidopsis* and tobacco because of lack of peach transformation system. Thus, our newly developed transgenic system will advance functional genomics studies and application of bioengineering for improvement of economically important traits in peach. Currently, stable transformation systems in perennial fruit trees, such as plum^43^ and apple^44^, not only require extensive experience and highly developed technical skills, but are also time-consuming and labor intensive, resulting a low transformation efficiency. The *A. rhizogenes*-mediated transformation system may be an option to overcome such shortcoming to conduct gene function analysis in fruit trees.

To date, most stable transformation systems were established based on *A. tumefaciens* in plants. In peach, one report of developing transgenic plants expressing the *GFP* gene using *A. tumefaciens*-mediated transformation system^5^. In this report, embryo-section was proved to be ideal explant for genetic transformation, while no plant generation or extremely low transformation efficiency were observed for hypocotyl and cotyledon explants. Besides *A. tumefaciens*, *A. rhizogenes* is also widely utilized to infect various explants, such as hypocotyl, leaf, stem, shoot and cotyledon, to induce transgenic tissues and/or plants^20,45^. Here, our study also showed that all tested explants, including hypocotyl, leaf and shoot, are suitable for *A. rhizogenes*-mediated transformation. Therefore, it seems that *Agrobacterium*-mediated transformation system is not tissue specific, and explants can be easily obtained. Although there are thousands of apple varieties, only cv. Gala shows relatively high transformation efficiency^x^. Similarly, our result showed that peach transformation efficiency was also affected by genotypes. Hence, identification of ideal genotype(s) may be a crucial step to establish stable plant transformation system in peach.

Callus tissues have an advantage of vigorous growth and can be easily collected without season and space limitation. Callus transformation system has been proven to be a rapid and efficient approach to assess gene function in fruit trees such as apple^46–49^ and citrus^50^. In peach, callus tissues induced from various explants such as leaf, stem and calyx have been reported, but there is no report on establishment of callus transformation system. Here, our study shows that transgenic hairy roots can be easily used to induce transgenic callus tissues, which not only represents an efficient way for long-term preservation of transgenic materials, but also provides an alternative approach to conduct gene function analysis.

One of the attractive features of *A. rhizogenes*-induced hairy roots is the ability to grow vigorously without supply of exogenous plant growth regulators^51^. This feature allows to produce composite plants through infecting shoots with *A. rhizogenes* to induce transgenic adventitious hairy roots^45^. In this study, both shoots and hair rooots of composite plants show a vigorous growth, suggesting that transgenic hair roots function properly. In fruit trees such peach, lack of rooting competence is one of the most crucial factors that limit clonal propagation of elite genotypes^52,53^. The composite plant system can be utilized as a biotechnology method for peach clonal propagation by directly inducing adventitious hairy roots from shoots. In addition, the composite plant system can serve as a platform for analysis of gene function, which allows for “in root” testing of transgenes in the context of a complete plant. Indeed, the composite plant system has been applied in a variety of gene function analyses and plant-microbe interaction studies^30,54–56^.

*PpMYB10* is a critical regulator that controls transcription of genes involved in anthocyanin biosynthetic pathway, and its biological function has been testified via transient expression system^35,57,58^. In this study, stable transformation system was used for the first time to validate the function of *PpMYB10.1* in transgenic roots of composite plants, and anthocyanin pigmentation was detected in transgenic hairy roots. Among anthocyanin biosynthetic pathway genes, only *PpUFGT* was highly activated by *PpMYB10.1*. This suggests that *PpUFGT* encodes a rate-limiting enzyme that determines the overall rate of anthocyanin biosynthesis, which supports the previous finding of *PpMYB10.1* activating *UFGT* in tobacco^57^. Besides *PpUFGT*, an anthocyanin transporter gene *PpGST* was also significantly activated, which is consistent with previous reports that *GST* acts downstream of anthocyanin-related *MYB* gene *MYB10.1*^59,60^. These results demonstrate that our newly developed transgenic root system has applications in functional characterization of genes and genetic engineering in peach or its related fruit trees of the Rosaceae family.

Our study developed a stable and reproductive *A. rhizogenes*-mediated transformation method to generate transgenic hairy roots and composite plants in peach. This method provides an efficient way to assess gene functions, genetic engineering, and root-rhizosphere microorganism interaction in peach.

## Materials and Methods

### *A. rhizogenes* strain and binary vector

Binary vectors pMV2G with 35S::DsRED1 cassette^28^ and pSAK277 containing *PpMYB10.1* ORF under the control of CaMV35S promoter^35^ were individually introduced into *A. rhizogenes* strain by heat shock transformation. Transformants were selected on YT plates containing 50 mg/L spectinomycin or 50 mg/L kanamycin. The single clone of positive *Agrobactrium* stain with the binary vector was inoculated in 1 mL YT liquid medium and then incubated at 28 °C with agitating at a speed of 180 rpm for 24 h. Approximately 500 μL of cell cultures were mixed with 300 μL of sterile glycerol and then stored in −80 °C until use.

*A. rhizogenes* from a glycerol stock were streaked on YT agar medium containing spectinomycin or kanamycin (50 mg/L), and then incubated at 28 °C for 2d. A single colony was transferred into a sterile tube containing 5 mL of YT broth plus spectinomycin or kanamycin (50 mg/L) and incubated at 28 °C with shaking at 180 rpm for 1d. Approximately 100 μL of *A. rhizogenes* cultures was transferred into a sterile 250 mL flask containing 100 mL YT medium (20 mg/mL acetosyringone) and incubated at 28 °C with shaking at 180 rpm for 12 h. Cell cultures were centrifuged at 700 g for 5 min and supernatant was discarded. Agrobacterium cells were resuspended in 1/2 MS to obtain final culture densities with OD_600nm_ values of 0.5, 0.8 or 1.2, respectively.

### Plant material and growth condition

Three peach varieties, ‘Shantao’, ‘Shengli’, and ‘Lvhuajiuhao’, used in this study are maintained in Wuhan Botanical Garden of Chinese Academy of Sciences, Wuhan, Hubei province. Peach pits were obtained from fully mature fresh fruit by peeling off the flesh with a knife and immersed in 3% sodium hypochlorite for 2 h to remove the residual flesh that staining on the surface of peach pits. The clean peach pits were dried under room temperature and stored at 4 °C for 45 days to reach the chilling requirement for germination. Subsequently, seeds without testa were surface-sterilized in 70% ethanol for 1 min and then in 3% sodium hypochlorite for 30 min, followed by washing with sterile distilled water for 6 times and soaked overnight at room temperature to increase germination efficiency. The treated seeds were placed on MS medium with 0.8% agar and incubated at 25 ± 1 °C in dark for 5 days and then transferred into light condition for three weeks. Seedlings with fully expanded leaves were used for genetic transformation.

### Explant preparation and transformation

One-month-old healthy seedlings with fully expanded leaves were used for transformation. After removing leaves and roots, hypocotyls were cut into 1-cm segments using a sterile scalpel, and were immersed in the *A.rhizogens* strain MSU440 suspension with control plasmid or recombined plasmid of interest genes at room temperature. According to the previous report^27,32^, co-transformation efficiency was assessed with different incubation time (10, 20, 30, or 40 min) (infection concentration OD_600nm_ = 0.8) and different concentration of *A. rhizogenes* suspension (OD_600nm_ = 0.5, 0.8, or 1.2) (30 min incubation duration). Hypocotyl segments were then transferred onto the filter paper to remove the redundant suspension, and placed onto the MS agar medium containing acetosyringone (20 mg/L) for co-cultivation in dark at 23 °C for 3 d. After co-cultivation, hypocotyl segments were washed with sterile distilled water containing cephalosporin (400 mg/L), and transferred onto fresh MS medium containing 400 mg/L cephalosporin and 50 mg/L spectinomycin or kanamycin, and incubated at 25 ± 1 °C with a 16 h photoperiod.

### Production of composite plants

One-month old healthy seedlings with fully expanded leaves were used for composite plants production. Seedlings were cut in the middle of the internode region to remove roots. Shoots that were ready for *A. rhizogenes* infection were maintained in sterile water to prevent withering. During the process of *A. rhizogenes* infection, shoots were placed into the plastic tray with apertures of 0.5 cm in diameter, and the cut sites of shoots were immersed into *A. rhizogenes* suspension in a plastic box. Shoots were shaken every 10 min to increase infection efficiency. After infection for 30 min, the plastic tray containing shoots was washed three times by immerging in sterile water. The infected shoots were then transferred onto the co-culture MS medium and incubated at 25 ± 1 °C under darkness for 3 d. Subsequently, cut sites of shoots were placed into the sterile plastic tray and washed three times with sterile water containing cephalosporin (400 mg/L). Infected shoots were put into the fresh MS medium containing 400 mg/L cephalosporin and 50 mg/L spectinomycin or kanamycin, and then incubated at 25 ± 1 °C with a 16 h photoperiod for hairy roots regeneration.

### Fluorescence assay and PCR analysis of regenerated hairy roots

The transgenic hairy roots were confirmed by fluorescence of DsRED1 protein and PCR using *DsRED1, rol* B, and *vir* D specific primers. Three-week-old regenerated hairy roots were visualized to detect DsRED1 fluorescence signal using fluorescence stereomicroscope equipped with a digital camera (SMZ25, Nikon). Fluorescence signals were observed using 400 nm excitation filter and 600 nm emission filter. PCR analysis of regenerated roots was conducted using Plant direct PCR kit following manufacturer’s instructions (Vazyme, PD105). Briefly, fresh roots were ground in the lysis buffer and then incubated at 95 °C for 10 min. Approximately 2 μL of the suspension was used as template for PCR analysis. Primers sequences are shown in Table S1.

### RNA extraction and real-time PCR (qRT-PCR) analysis

Total RNA was extracted using Total RNA Rapid Extraction Kit (ZOMANBIO, Beijing, China) following manufacturer’s instructions. First-strand cDNA synthesis was conducted using PrimerScript^TM^RT reagent Kit with gDNA Eraser (Takara, Dalian, China). Quantitative RT-PCR was performed in a total reaction volume of 20 μL containing 0.4 μM of each primer, 1 × ROX reference dye, 10 μL of 2 × SYBR premix Ex Taq II (TaKaRa), and 100 ng of template cDNA. The program for qRT-PCR was as follows: one cycle of 30 s at 95 °C, followed by 40 cycles of 5 s at 95 °C and 34 s at 60 °C. All analyses were conducted with three biological replicates. The primers used for quantitative RT-PCR are listed in Table S1.

## Supplementary information

Supplementary Information.
