# Supplementary material for: Development of a fast and efficient root transgenic system for functional genomics and genetic engineering in peach
Source: Sci Rep. 2020 Feb 18;10:2836. doi: 10.1038/s41598-020-59626-8 (PMC7029003; doi:10.1038/s41598-020-59626-8)
Supplement: Supplementary file 1 — Supplementary Information. [file 41598_2020_59626_MOESM1_ESM.pdf]

Supplementary Information

Development of a fast and efficient root transgenic system for functional genomics and genetic engineering in peach

Shengli Xu[1,2¶], Enhui Lai[1,2¶], Lei Zhao[1,2], Yaming Cai[1,2], Collins Ogutu[1,2], Sylvia Cherono[1,2], Yuepeng Han[1,3*], Beibei Zheng[1*]

Table of contents

Table S1 List of primers used in this study
Figure S1 Expression levels of genes involved in anthocyanin synthesis in transgenic and wild type roots.
Figure S2 The full-length gels for cropped gels used in Figure 2 (a-c), 3 (d) and 5 (e-g).

Table S1 List of primers used in this study

gene	Primers (5'-3')	
	Forward	Reverse
PpMYB10.1	GAAATGATTGGTGGGAAACC	GTCCTTCTTCTGAAACATTGGT
PpGST	ACTTCAACTTCTGGTGCTGC	GCTGCTCTTTGATAATCTTTCC
PpUFGT	GTCCTAGCCCATGCTTCAGTC	GATCTCCAACACGTCCTCCAC
PpGAPDH	TGCCATTGAAATCCTGAAAC	ACCAATTGGATCATCCTCCT
PpCHS	*AACAAGGGTGCTCGTGTTCTC*	*GCTGCACCATCACCGAATAAG*
PpCHI	*GAGATCGTTACAGGTCCATTTG*	*GTGGGAAGTTTTGATCCTTGA*
PpF3H	*GGACTGGACACAGAGGCATT*	*AATTGTGCCTGGGTCAGTGT*
PpF3'H	*CTCTCGCTCAAAGAGGATGC*	*CCATTCCACTGTGCTTGATG*
PpDFR	*CGCCTCCAAGACTCTAGCTG*	*CCAGTGAGTGGGGAAAGTCC*
PpLDOX	*AGGAGTTGAAGAAGGCAGCA*	*GCCTGGTCATTGGCATACTT*
DsRED1	CCCAGTTCCAGTACGGCTC	*ATGGTGTAGTCCTCGTTTGTG*
PpMYB10.1-PSAK277	ACGCACAATCCCACTATCCT	TCATGCGATCATAGGCGTCT
rolB	GCTCTTGCAGTGCTAGATTT	GAAGGTGCAAGCTACCTCTC
VirD	ATGTCGCAAGGCAGTAAG	CAAGGAGTCTTTCAGCATG

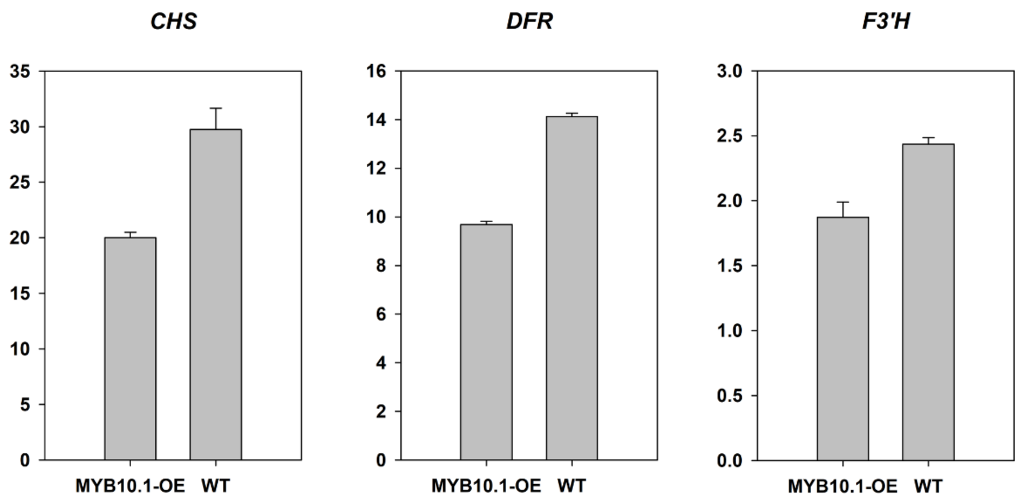

Figure S1 Expression levels of genes involved in anthocyanin synthesis in transgenic and wild type roots.

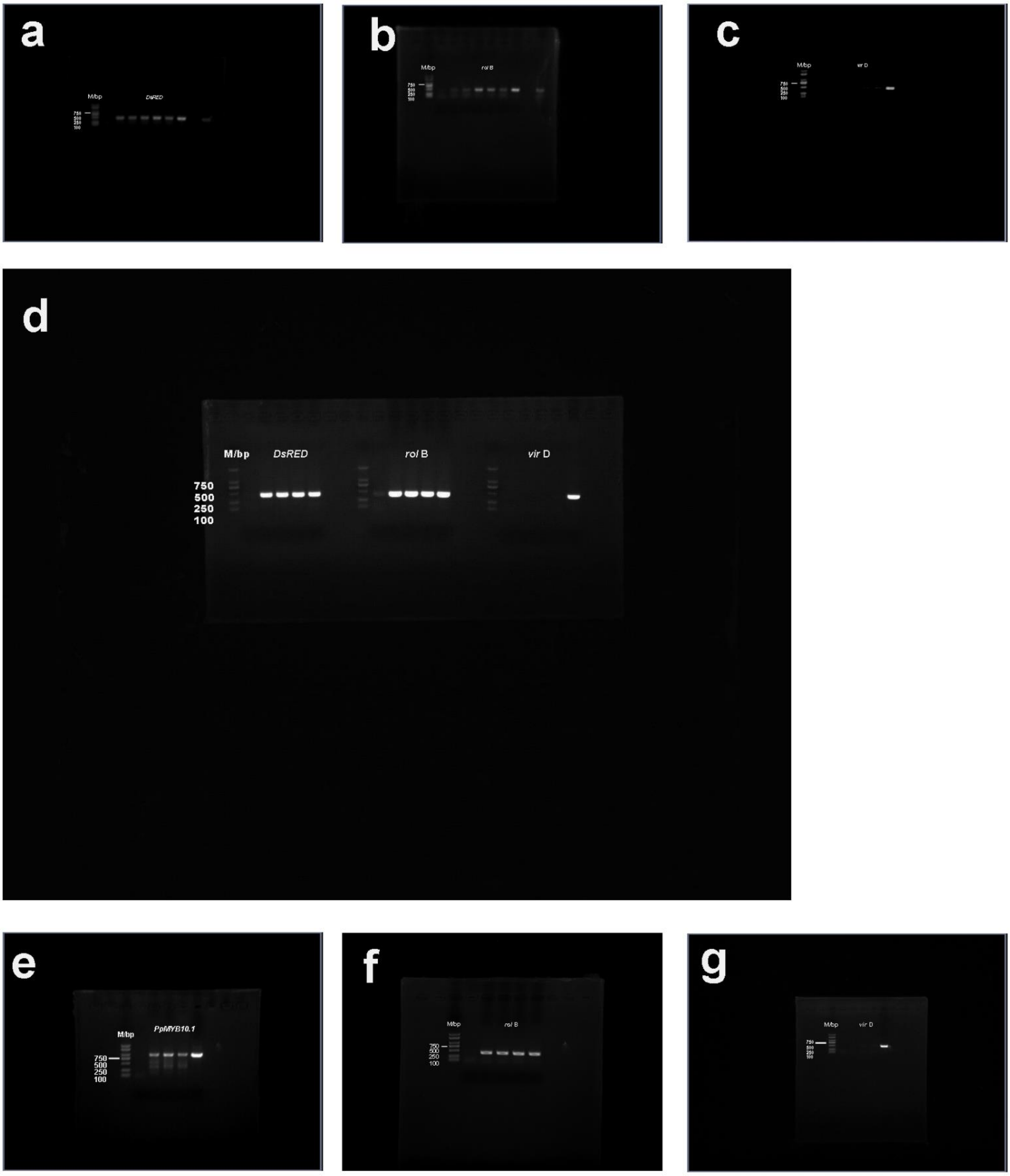

Figure S2 The full-length gels for cropped gels used in Figure 2 (a-c), 3 (d) and 5 (e-g).
